# Hoarding Disorder and Diogenes Syndrome: Two Case Reports and a Narrative Review

**DOI:** 10.7759/cureus.78289

**Published:** 2025-01-31

**Authors:** Andreia Certo, Odete Nombora, Tatiana Pessoa, Rita Neto, Eva Mendes

**Affiliations:** 1 Psychiatry Department, Vila Nova de Gaia/Espinho Hospital Centre, Vila Nova de Gaia, PRT

**Keywords:** dementia, diogenes syndrome, hoarding disorder, obsessive-compulsive disorder, squalor

## Abstract

Hoarding disorder (HD) and Diogenes syndrome (DS) are challenging and multifaceted conditions that significantly impact patients and society. These disorders are characterized by excessive hoarding behaviors, often accompanied by multiple physical and mental comorbidities, diminished quality of life, and substantial public health challenges. Through the presentation of two case reports and a narrative review, this article revisits HD and DS, highlighting the need for clinical awareness, targeted management strategies, and further research in this field. The first case report describes a 35-year-old woman with early-onset HD and comorbid obsessive-compulsive disorder, while the second details a 78-year-old woman with DS related to vascular dementia. These cases underscore the necessity of timely diagnosis, multidisciplinary interventions, and comprehensive management to prevent deterioration, address physical health issues, improve hygiene and home safety, and mitigate harm to both patients and the community. Advanced treatments such as transcranial magnetic stimulation and electroconvulsive therapy may also play a role in managing these complex conditions. The development of practical guidelines is essential for the effective assessment and care of patients with such multidimensional needs. Moreover, there is a critical need for well-designed randomized controlled trials to evaluate and compare pharmacological and non-pharmacological treatment approaches.

## Introduction

Hoarding of objects has increasingly become a significant public health hazard due to its widespread consequences, including safety risks such as fire hazards and structural damage, sanitation problems that lead to health risks like infections and infestations, pressure on resource allocation, and ethical challenges involved in the management and treatment of these cases [1,2]. Hoarding is characterized by the excessive acquisition and collection of items, often of little or no value, to the point of interfering with an individual's living conditions and daily functioning [3,4]. This condition can range from a benign habit to a pathological behavior, and it is essential to distinguish between the various disorders that feature hoarding as a characteristic behavior [5].

Hoarding disorder (HD) was recognized as a distinct diagnostic entity in the fifth version of the Diagnostic and Statistical Manual of Mental Disorders (DSM-5) [6,7] and the International Classification of Diseases 11th Revision [8]. In cases with early onset, hoarding is often associated with psychiatric disorders such as obsessive-compulsive disorder (OCD), mood or anxiety disorders, psychotic disorders, and alcohol use disorder [6,7,9]. However, in the elderly, hoarding tends to present as an isolated syndrome, known as primary Diogenes syndrome (DS), or may be associated with neurodegenerative diseases such as dementia or other mental disorders [6,7,9]. Some researchers believe that HD and DS are interconnected and should be viewed as part of a clinical continuum [6].

This article presents two case reports that illustrate different manifestations of these conditions: the first involves a 35-year-old woman with HD comorbid with OCD, and the second involves a 78-year-old woman with DS due to vascular dementia. Through these case reports, we aim to highlight the importance of early diagnosis, clinical awareness of these conditions, and the need for multidisciplinary treatment approaches.

## Case presentation

First case

We describe a case of a 35-year-old woman, married, living with her husband and daughter, unemployed, who was brought to the emergency room due to excessive hoarding of trash and other possessions, along with aggressiveness when her family tried to clean the house. Her possessions included litter, food, and several items, all neatly organized and labeled, which were part of her own categorization system. She presented herself well-dressed and clean, with a history of depressive symptoms and a diagnosis of severe OCD from early adolescence, characterized by ruminative doubts, obsessive thoughts, and repeated checking behaviors.

The hoarding behavior began at the age of 25, after the birth of her daughter, when she felt unable to properly care for her home and family. Ten years later, the situation worsened after she lost several valuable items during a move to a new house. She became convinced that certain items had been discarded in the trash and began to check all garbage bags to ensure that nothing valuable was thrown away.

She became a recluse at home, fearing that her family would clean out her belongings. Although she recognized the behavior as problematic, she justified it by saying that every item could be useful someday, including the trash. In addition, she reported not taking her psychiatric medication (fluvoxamine 200 mg/day) because “If I take it, I will not be able to hoard and organize things anymore” (sic).

The patient was admitted several times to a psychiatric inpatient unit to allow the cleaning of her home. During her hospitalization, she tended to accumulate trash in her purse and her bedroom closet, showing anxiety about the possibility of cleaning her house. At the time, the patient was already taking clomipramine 50 mg/day. The dose was gradually titrated to 150 mg/day, and she became stabilized after three weeks. Ten years later, following a divorce, she moved into her mother’s house. Throughout this period, she continued on clomipramine 150 mg/day but could not afford other therapeutic approaches, such as psychotherapy. In her mother’s house, a more structured environment, she did not hoard again, although the desire to do so persisted.

Second case

We describe a case of a 78-year-old woman, widowed for over 40 years and living alone, who was admitted to the emergency room due to agitation and aggressiveness after her relatives attempted to enter her home. She was living in a filthy environment, hoarding trash and worthless objects in a disorganized and random way. In several parts of the house, the floor was completely covered with items, preventing passage between rooms. She was self-neglected, wearing malodorous clothing, and had isolated herself from her family and the community, refusing any form of help. This behavior began four years earlier, after a hemorrhagic stroke due to an aneurysm rupture in the anterior cerebral artery. At the time, she was submitted to an emergency surgery. Her family noticed that she started to isolate herself, refusing to leave the house or accept visitors, including close relatives.

**Figure 1:**
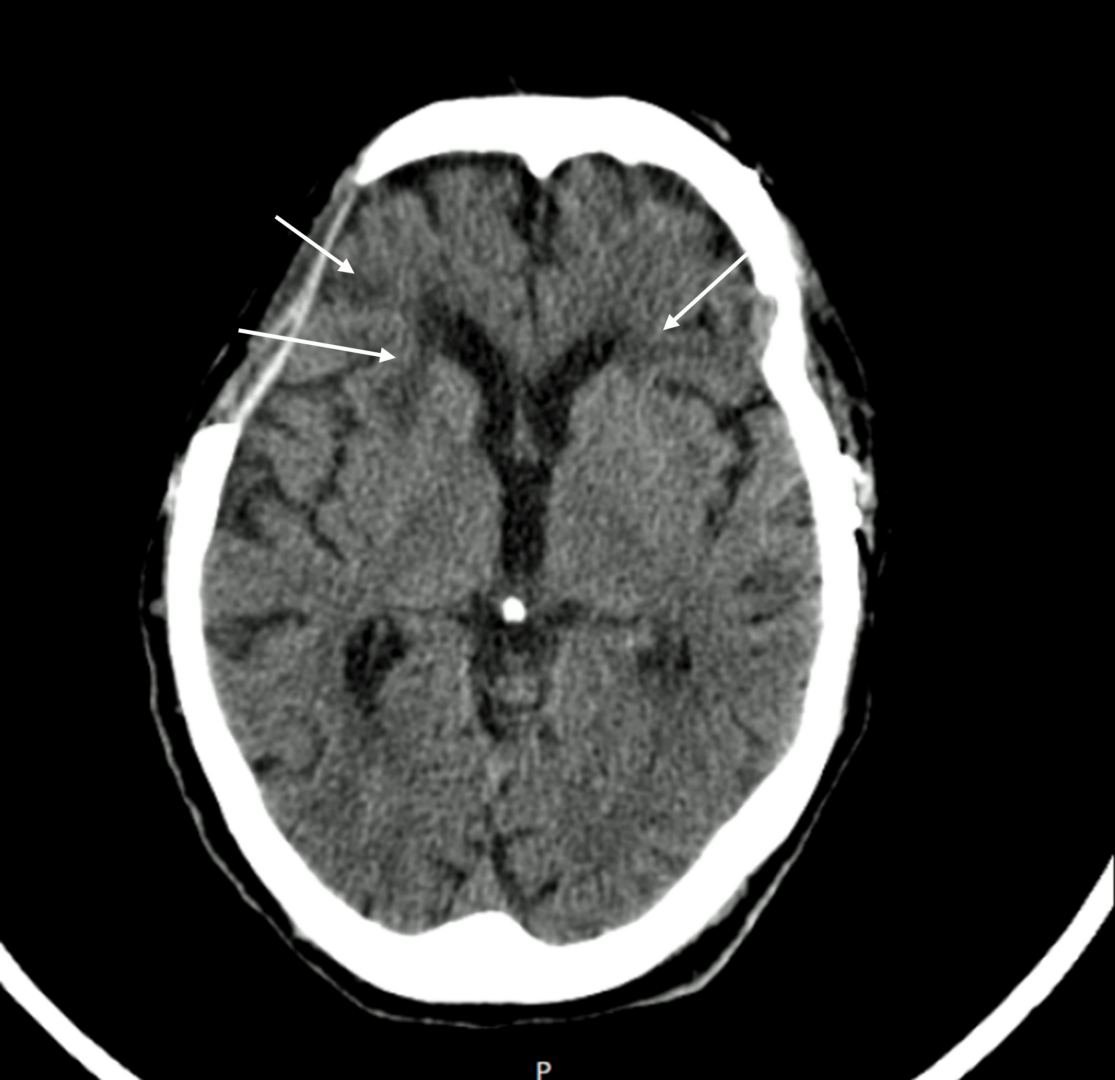
Cerebral CT scan Signs of surgical approach via left fronto-parieto-temporal craniotomy and right fronto-parieto-temporo-pterional craniotomy. Bilateral ischemic cortico-subcortical lesions in the fronto-basal and anterior temporal and periventricular regions.

The patient was involuntarily admitted to a psychiatric facility. During her stay, she was disoriented in time and space and exhibited circumstantial speech, mystic delusions, and paranoid ideas about her neighbors, with no insight into her condition. A cerebral computed tomography (CT) scan was performed and revealed several vascular lesions related to the hemorrhagic stroke (Figure 1). She was then diagnosed with vascular dementia and was unable to live alone or take care of herself. She was stabilized with risperidone 2 mg/day and lorazepam 1 mg three times a day. Upon discharge, the patient was transferred to a senior residential institution as her family was unable to take care of her at home.

## Discussion

Hoarding disorder

The first case presented highlights an HD with early-age onset in the context of OCD.

The term "hoarding" is a complex concept that has been extensively discussed over the years. As a psychopathological phenomenon, it was first described by Bolman and Katz in the 1960s and has since been reported in a variety of psychiatric disorders, ranging from OCD to schizophrenia. Several theoretical approaches have been proposed to understand hoarding behaviors, including psychoanalytical to functional relationships between cognitions and behaviors. In 1996, Frost and Hartl suggested a widely employed cognitive-behavioral model of hoarding, which conceptualizes the behavior as a consequence of 1) information-processing deficits; 2) problems in forming emotional attachments; 3) behavioral avoidance; and 4) erroneous beliefs about the nature of possessions. These authors also suggested that clinical compulsive hoarding should be defined as (1) the acquisition of, and failure to discard a large number of possessions that appear to be useless or of limited value; (2) living spaces sufficiently cluttered so as to preclude activities for which those spaces were designed; and (3) significant distress or impairment in functioning caused by the hoarding. Several features of this definition are noteworthy [8]. 

Historically, HD was considered a subtype of OCD. As mentioned before, based on the differences between hoarding and OCD, the DSM-5 classified HD as a discrete condition (Table 1) [8,10].

**Table 1:** Diagnostic and Statistical Manual of Mental Disorders (DSM-5) hoarding disorder - abbreviated diagnostic criteria

Disorder class: obsessive-compulsive and related disorders
A. Difficulty discarding or parting with possessions, regardless of their value.
B. This difficulty is due to a perceived need to save the items and to distress associated with discarding them.
C. The difficulty of discarding possessions results in the accumulation of possessions that congest and clutter active living areas and substantially compromise their intended use.
D. The hoarding causes clinically significant distress or impairment in social, occupational, or other important areas of functioning.
E. The hoarding is not attributable to another medical condition.
F. The hoarding is not better explained by the symptoms of another mental disorder.

While determining the exact prevalence of hoarding with early-age onset is challenging, it is estimated that 11-42% of OCD patients have this condition [5], and the overall prevalence ranges from 1.5% to 5.8% [2]. HD typically emerges during adolescence, develops at 20-30 years of age, and has a chronic and progressive course, and commonly, there is a family background of this condition [2,5]. It is associated with several physical health issues, a diminished quality of life, and higher dysfunction [2]. Interestingly, patients with this condition usually do not show signs of self-neglect and tend to have an earlier onset of the condition [6]. Furthermore, they often exhibit other obsessive features and experience distress if they are unable to hoard objects [2,6].

Young patients with hoarding often accumulate bizarre or unusual items, storing them at home [5,11]. Although the hoarding may appear chaotic, there is usually a personal, idiosyncratic order, with items grouped in categories that may appear disorganized to outsiders [5,11]. Over time, the accumulation of possessions fills the home, and the individual struggles to discard or remove unnecessary items [5,11]. Initially, these patients may deny or downplay their hoarding behaviors, attempting to justify it [5,11]. This egosyntonic presentation often manifests in patients defending their actions by claiming that there is a practical reason for keeping their possessions, particularly with regard to their potential future usefulness [5,8,11]. The patients tend to experience anxiety if they are unable to hoard items and often react with anger or aggression if someone changes how their objects are stored, seeing it as a violation of their need for control [2,5,8,11]. This is a central feature of the disorder [2]. Interpersonally, they often view their possessions as extensions of their identity, with the objects providing them with a sense of control and security [5,11]. They may also develop other obsessive behaviors, such as compulsive cleanliness rituals or impulsive phobias [5,11]. On the other hand, hoarding-related compulsions tend to provide a sense of pleasure and reward and are often worse over time, particularly with each passing decade [8].

Cognitive functions in individuals with HD are generally preserved, although they tend to display a cognitive pattern reflective of obsessive traits, such as difficulty prioritizing the importance of things and an overwhelming desire for safety and control over unforeseen situations [5]. It is often linked to personality traits like perfectionism, indecisiveness, and procrastination [2]. Hoarding behavior tends to be secretive, often limited to the home environment, and social functioning is typically maintained, except in severe cases [5,12].

Patients with HD usually seek psychiatric help due to personal life deterioration rather than recognizing their hoarding behavior as a problem, even when they are aware of its negative consequences [5].

Differentiating HD from other disorders is essential. For example, unlike collecting behaviors in OCD, in HD, the hoarding behavior typically involves limited insight, lack of resistance to compulsions, and low motivation for treatment. In addition, hoarding often has poor responses to standard treatments, including selective serotonin reuptake inhibitors (SSRIs) and cognitive-behavioral therapy (CBT) [8]. By contrast, collecting in OCD is driven by obsessions and is usually associated with anxiety [8]. The main distinction between HD and DS is that the latter is characterized by self-neglect, squalor, and a lack of insight, distress, and emotional attachment [3]. It is also important to differentiate HD and collectionism, an organized and systematic activity, in which the objects are kept in specific and structured places, with the purpose of organizing and hierarchizing objects, instead of hoarding them and the items are frequently valued by other collectors, and they like to show them as well [5,11,12].

Diogenes syndrome

The second case discusses an elderly patient with DS secondary to vascular dementia.

DS, also referred to as severe domestic squalor [12], is characterized by social withdrawal, extreme self-neglect, pathological hoarding of various objects/trash, lack of concern for one's living condition, and rejection of external help [4,6,7].

The condition was first described by Dupré in 1925 and later by Stevens in 1963, who described the first cases of severe domestic squalor in the elderly [3,6]. The term “Diogenes syndrome” was coined by Clark, Mankikar, and Gray in 1975 [3,6]. It was inspired by a Greek philosopher named Diogenes the Cynic (412-323 BC) who had the features that characterize the syndrome [6,7]. It is a relatively rare disorder, although its true incidence is underreported, as many patients do not seek help and are often noticed by neighbors, relatives, or social workers [6]. A study shows that the annual incidence of DS is around 0.05% among individuals over the age of 60, with the majority being single or widowed and living alone [13].

DS is considered a life-threatening condition, as it often goes undetected until the patient faces medical collapse due to their resistance to services [1,6,7]. Extreme self-neglect manifests in poor hygiene, inadequate food intake, and disregard for health, while patients typically reject any type of assistance and experience isolation, showing no shame about their living conditions [6,11]. Unlike hoarding behavior in HD, there is no order or emotional attachment to the possessions in DS, and patients tend to deny or minimize the problem, as well as to rationalize it [5,7].

DS is usually observed in those over 65 years, and even though its etiology is unclear [14], it has been linked to psychiatric disorders such as schizophrenia, bipolar disorder, and depression [3,6,9,11,12,14], personality traits like obsessive-compulsive, avoidant, paranoid, schizotypal and antisocial [11,12,14], childhood adversity, dementia, especially frontotemporal dementia [6,7,12], damage of prefrontal areas of the brain [15,16], and a history of alcohol dependence [6,7,9] or other substance abuse and physical disability [12]. Some of these patients have no psychiatric disease at all [5,6].

It has been hypothesized that DS results from deficits in decision-making, potentially related to impaired functioning of the orbitofrontal, mediofrontal, and anterior cingulate networks, with serotoninergic and dopaminergic neurotransmission dysfunction playing a role [6,9].

In summary, the following table outlines the key differences between HD and DS (Table 2).

**Table 2:** Summary of differences between hoarding disorder and Diogenes syndrome

Feature	Hoarding disorder	Diogenes syndrome
Onset	Adolescence or early adulthood	Typically, after age 65
Insight	Limited insight, often aware of consequences	No insights, denies the severity
Emotional attachment	Strong attachment to possessions	No attachment, often cluttered or trash-filled
Self-neglect	Not common	Extreme self-neglect, poor hygiene
Living conditions	Cluttered but functional	Squalor, unsafe living conditions
Social withdrawal	Generally, maintains social relationships	Severe social withdrawal, rejects help
Cognitive function	Generally intact, obsessive traits present	Often impaired, especially in dementia

Assessment and management of HD and DS

Assessing HD and DS requires a multidisciplinary approach. Information about the environment, symptom severity, comorbidities, functional status, and safety risks should all be considered [2].

In hospital settings, DS should be suspected if the patient presents severe squalor, such as layers of soiled clothing. Reports from relatives, neighbors, and social workers are valuable, and a home visit is essential to confirm the diagnosis [6,7]. In addition, screening tools like the “Environmental Cleanliness and Clutter Scale” developed by Halliday and Snowdon can be used to assess squalor and hoarding behavior [6,7,12].

Management of HD or DS includes both pharmacological and non-pharmacological interventions, as well as addressing the underlying physical and mental health comorbidities [2,3,6,7,9]. Pharmacotherapy often depends on the underlying symptoms and psychiatric disorders and seems to be a challenge [6]. A review of four pharmacotherapy studies suggested improvement in HD with medications such as paroxetine, venlafaxine, methylphenidate, and atomoxetine [2], while another review, which included clinical cases, suggested that risperidone, quetiapine, valproic acid, and lithium may show greater efficacy in managing DS [6]. However, the response to psychopharmacology treatment is not consistent [7]. Behavioral approaches, such as CBT adapted to HD (CBT-H) and motivational strategies, have shown promising results, focusing on the key components of compulsive hoarding [2,6,7,12]. In addition, some studies have shown that transcranial magnetic stimulation and electroconvulsive therapy may be effective for obsessive-compulsive-related disorders [17,18]. Social strategies and multidisciplinary approaches are highly emphasized in the management of these conditions [2,3,6,12].

Treatment often requires hospitalization, institutionalization, moving the patient to another accommodation, or community outpatient treatment [6,7], with a case management approach [12]. It is essential to address treatment with sensitivity, building trust with the patient in order to prevent relapses and reduce treatment resistance [3,6,7,12].

Prognosis depends on the individual’s ability to reintegrate into society, although clinical relapses are common, and poor physical health and early age of onset are adverse prognostic factors [19]. Due to isolation and neglect, many patients are discovered post-mortem [2,20].

The management of these patients presents medical, ethical, and legal challenges, ranging from forced removal from their homes to intervention by health authorities due to public health risks [1,6].

## Conclusions

These two cases have been reported to raise clinical awareness and highlight that individuals living in pathological hoarding conditions may suffer from psychiatric or neuropsychiatric disorders. Differentiating between HD and DS is crucial, as this distinction could pave the way for more advanced research and the development of more effective pharmacological and non-pharmacological interventions.

It is essential to emphasize the importance of timely diagnosis, early multidisciplinary interventions, and comprehensive management strategies for these patients. Such approaches are vital not only to prevent the progression of physical illnesses but also to improve personal and home hygiene. They can also ensure safety and reduce harm to both patients and the community, contributing to better public health outcomes. Therefore, the establishment of practical guidelines for the appropriate assessment and management of these patients with complex, multifaceted needs is urgently needed. In addition, neurostimulation methods, such as electroconvulsive therapy and transcranial magnetic stimulation, may also play a role in managing these complex conditions and could be considered treatment options. Furthermore, there is a need for well-designed randomized controlled trials, to compare the effectiveness of pharmacological and non-pharmacological treatments.

## References

[1] Bonci G, Varghese E, Mahgoub N (2012). A case of Diogenes syndrome: clinical and ethical challenges. J Am Geriatr Soc.

[2] Gleason A, Perkes D, Wand AP (2021). Managing hoarding and squalor. Aust Prescr.

[3] Proctor C, Rahman S (2021). Diogenes syndrome: identification and distinction from hoarding disorder. Case Rep Psychiatry.

[4] Zuliani G, Soavi C, Dainese A, Milani P, Gatti M (2013). Diogenes syndrome or isolated syllogomania? Four heterogeneous clinical cases. Aging Clin Exp Res.

[5] Guillermo L, Dolores SG, Martín-Ballesteros E (2006). Differential diagnosis of hoarding behaviors. Actas Esp Psiquiatr.

[6] Assal F (2018). Diogenes syndrome. Front Neurol Neurosci.

[7] Sacchi L, Rotondo E, Pozzoli S (2021). Diogenes syndrome in dementia: a case report. BJPsych Open.

[8] Fontenelle LF, Grant JE (2014). Hoarding disorder: a new diagnostic category in ICD-11?. Braz J Psychiatry.

[9] Nath S, Saraf A, Pattnaik JI (2019). An extreme form of elder self-neglect: revisiting the Diogenes syndrome. Asian J Psychiatr.

[10] American Psychiatric Association (2013). Diagnostic and statistical manual of mental disorders 5th ed (DSM-5). https://www.psychiatry.org/psychiatrists/practice/dsm.

[11] Sansone RA, Sansone LA (2010). Hoarding: obsessive symptom or syndrome?. Psychiatry (Edgmont).

[12] South Australian Department for Health and Ageing; Health Protection Programs (2013). A foot in the door : stepping towards solutions to resolve incidents of severe domestic squalor in South 
Australia / South Australian Department for Health and Ageing; Health Protection Programs. A foot in the door - stepping towards solutions to resolve incidents of severe domestic squalor in South 
Australia.

[13] Cipriani G, Lucetti C, Vedovello M, Nuti A (2012). Diogenes syndrome in patients suffering from dementia. Dialogues Clin Neurosci.

[14] Biswas P, Ganguly A, Bala S, Nag F, Choudhary N, Sen S (2013). Diogenes syndrome: a case report. Case Rep Dermatol Med.

[15] Funayama M, Mimura M, Koshibe Y, Kato Y (2010). Squalor syndrome after focal orbitofrontal damage. Cogn Behav Neurol.

[16] Greve KW, Curtis KL, Bianchini KJ, Collins BT (2004). Personality disorder masquerading as dementia: a case of apparent Diogenes syndrome. Int J Geriatr Psychiatry.

[17] Diefenbach GJ, Tolin DF, Hallion LS, Zertuche L, Rabany L, Goethe JW, Assaf M (2015). A case study of clinical and neuroimaging outcomes following repetitive transcranial magnetic stimulation for hoarding disorder. Am J Psychiatry.

[18] Dos Santos-Ribeiro S, de Salles Andrade JB, Quintas JN, Baptista KB, Moreira-de-Oliveira ME, Yücel M, Fontenelle LF (2018). A systematic review of the utility of electroconvulsive therapy in broadly defined obsessive-compulsive-related disorders. Prim Care Companion CNS Disord.

[19] Irvine JD, Nwachukwu K (2014). Recognizing Diogenes syndrome: a case report. BMC Res Notes.

[20] Byard RW (2014). Diogenes or Havisham syndrome and the mortuary. Forensic Sci Med Pathol.

